# Uncovering the Contrasts and Connections in PASC: Viral Load and Cytokine Signatures in Acute COVID-19 versus Post-Acute Sequelae of SARS-CoV-2 (PASC)

**DOI:** 10.3390/biomedicines12091941

**Published:** 2024-08-23

**Authors:** Brandon Compeer, Tobias R. Neijzen, Steven F. L. van Lelyveld, Byron E. E. Martina, Colin A. Russell, Marco Goeijenbier

**Affiliations:** 1 Artemis Bioservices B.V., 2629 JD Delft, The Netherlands; b.compeer@artemisbioservices.com (B.C.); b.martina@artemisbioservices.com (B.E.E.M.); 2 Department of Medical Microbiology, University Medical Center Amsterdam (UMC, Amsterdam), 1105 AZ Amsterdam, The Netherlands; c.a.russell@amsterdamumc.nl; 3 Department of Intensive Care Medicine, Spaarne Gasthuis, 2035 RC Haarlem, The Netherlands; t.r.neijzen@student.vu.nl; 4 Department of Internal Medicine, Spaarne Gasthuis, 2035 RC Haarlem, The Netherlands; s.van.lelyveld@spaarnegasthuis.nl; 5 Department of Intensive Care, Erasmus MC University Medical Centre, 3015 GD Rotterdam, The Netherlands

**Keywords:** SARS-CoV-2, COVID-19, PASC, long COVID, cytokines, viral load, upper respiratory tract, lower respiratory tract, plasma

## Abstract

The recent global COVID-19 pandemic has had a profound and enduring impact, resulting in substantial loss of life. The scientific community has responded unprecedentedly by investigating various aspects of the crisis, particularly focusing on the acute phase of COVID-19. The roles of the viral load, cytokines, and chemokines during the acute phase and in the context of patients who experienced enduring symptoms upon infection, so called Post-Acute Sequelae of COVID-19 or PASC, have been studied extensively. Here, in this review, we offer a virologist’s perspective on PASC, highlighting the dynamics of SARS-CoV-2 viral loads, cytokines, and chemokines in different organs of patients across the full clinical spectrum of acute-phase disease. We underline that the probability of severe or critical disease progression correlates with increased viral load levels detected in the upper respiratory tract (URT), lower respiratory tract (LRT), and plasma. Acute-phase viremia is a clear, although not unambiguous, predictor of PASC development. Moreover, both the quantity and diversity of functions of cytokines and chemokines increase with acute-phase disease severity. Specific cytokines remain or become elevated in the PASC phase, although the driving factor of ongoing inflammation found in patients with PASC remains to be investigated. The key findings highlighted in this review contribute to a further understanding of PASC and their differences and overlap with acute disease.

## 1. Introduction

The COVID-19 pandemic profoundly altered everyday life for an extended duration while claiming the lives of millions [1]. The spectrum of COVID-19 disease ranges from mild subclinical upper respiratory tract (URT) symptoms to acute respiratory distress syndrome (ARDS), with a high mortality. Although most infected individuals undergo a mild acute phase of illness and another large group recovers from more severe disease [2], numerous people have reported persistent symptoms following SARS-CoV-2 infection [3]. Those with Long COVID or Post-Acute Sequelae of COVID-19 (PASC) show pathological damage across diverse organs, including the nervous and immune system, blood vessels, and lungs. This damage manifests in a variety of long-lasting symptoms, most commonly including fatigue, respiratory issues, and neurological problems [3,4]. In an effort to characterize this multisystem disease, recent research has focused on phenotyping according to PASC symptom clustering, which may facilitate linking PASC phenotypes and their underlying mechanisms [5,6]. It should be noted that various terms within COVID-19- and PASC-related research are used, often with varying definitions. Therefore, the key terms used in this review are summarized in an explanatory box (Table 1) providing their definitions as used here. Several studies have investigated the viral load and release of cytokines and chemokines as key factors influencing COVID-19 disease progression and overall outcome. Nasopharyngeal samples of patients with severe disease often display high viral loads [7,8]. Furthermore, the plasma inflammatory markers interleukin (IL-) 6 [9], IL-10, and IL-2 [10] are increased in patients with severe acute phase disease. In the fight against the pandemic, both factors were predominantly studied considering short-term outcomes, including 30-day mortality and Intensive Care Unit (ICU) admission. There remains, however, a scarcity of reviews examining the correlation between viral loads in different organs and the release of cytokines and chemokines among various clinical subsets of COVID-19 disease. Although progress has been made regarding viral and immune dynamics during the acute phase of COVID-19, a clear virological perspective on the link between the cytokine profiles and viral load that dominate acute-phase outcome and subsequent PASC development remains missing. This challenge partly results from the need for long-term studies with extensive follow-up times. Moreover, the diverse symptoms [4] and recently emerging evidence of different PASC phenotypes [11,12] add to the complexity, as the inclusion of a significant number of persons with a diverse range of PASC symptoms is required. In addition, as Greenhalgh et al. [13] have clearly outlined, clinical phenotyping studies have utilized various methods on different samples, therefore leading to different PASC phenotype clusters [4,11,12,13]. Therefore, here, we review the current body of knowledge on the viral load and cytokine and chemokine release during the acute phase in COVID-19 patients. In addition, we summarize studies that have aimed to establish whether there is a correlation between viral load and increased release of cytokines and chemokines. Determining the existence of such correlations is important, as dysregulated immunity frequently leads to pathophysiological damage. Hence, particularly in hospitalized cases, anticipating heightened cytokine and chemokine release in patients with elevated viral load could be beneficial. Prophylactic medications that inhibit this release may mitigate pathophysiological damage. Finally, this review aims to explore the disparities and associations within cytokine and chemokine profiles among patients during acute disease and those experiencing PASC. This exploration may provide valuable predictive insights into PASC development.

## 2. Materials and Methods

We conducted a narrative examination of the current body of literature. The search engines PubMed and Google Scholar were searched for the following keywords, which were additionally combined as ad hoc strings for advanced search: COVID-19; SARS-CoV-2; viral load; disease severity; viremia; cytokines; chemokines; URT; upper respiratory tract; LRT; lower respiratory tract; BAL; serum; plasma; PASC; long COVID. First, relevant research and review articles were selected based on the title and abstract. Next, inclusion for the review was decided based on a thorough analysis of the content.

## 3. Viral Loads

### 3.1. Viral Loads during Acute COVID-19

**Figure 1:**
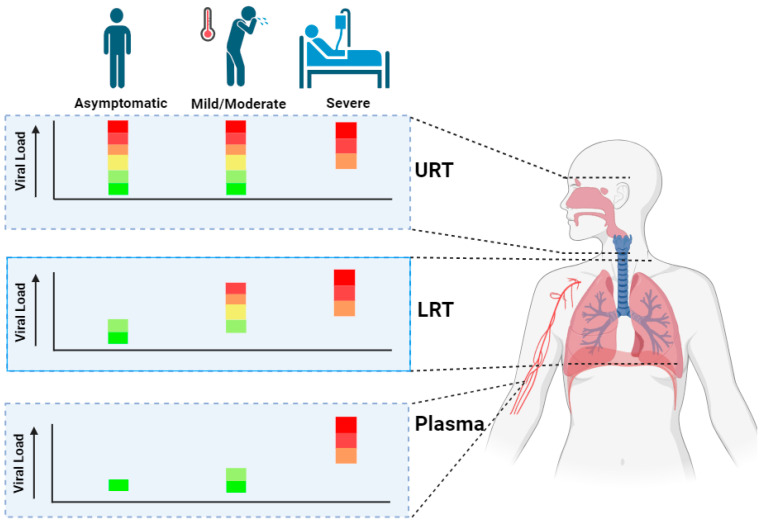
Graphical representation of viral loads observed across clinical classifications of acute-phase COVID-19 disease. Colored bar graphs illustrate viral load levels, ranging from red (high viral load) to green (low viral load). Viral load levels (low-high) are interpretated according to the perspectives of the authors referenced in cited articles. Graphical representation is based on the following references: [14,17,18,19,22,23,24,25,26]. Figure created using https://www.biorender.com/ (accessed on 12 March 2024).

Various studies have shown that the viral loads in the URT are unrelated to clinical presentation [14,15], while others have proposed a correlation [16]. Although distinct factors may explain this contradiction, including timing of sampling (e.g., pre or post symptom onset), differences in population heterogeneity, and outcome assessment, the connection between viral loads in the URT and disease severity is still a subject of debate. On the contrary, high URT viral loads in hospitalized, clinically severe patients were significantly correlated with higher risks of intubation, critical progression, and in-hospital mortality (Figure 1) [14,17,18,19]. Cytokine storms (Table 1) are more often observed in patients that display critical progression and, in some cases, mortality [20,21]. Given that cytokine storms are characterized by a dysregulation of both the pro- and anti-inflammatory immune response, this dysregulation may lead to loss of control on viral replication. Hence, we hypothesize that, in severe and critical cases, tipping points may be reached where the predisposition or occurrence of a cytokine storm triggers increased viral replication, consequently leading to higher URT viral loads.

While mild COVID-19 disease is usually confined to the URT, severe disease often manifests when viral infection reaches the lower respiratory tract (LRT) [27]. Therefore, a correlation between high LRT viral loads and disease progression is anticipated. This has been shown by Ynga-Durand et al., who found that the viral load in bronchoalveolar lavage (BAL) samples obtained from the LRT of severely ill patients admitted to the ICU were linked to fatal outcomes [22]. Furthermore, a retrospective study that analysed sputum obtained from hospital admitted mild-to-moderate (*n* = 62) or severe (*n* = 30) patients found that those with severe disease at baseline displayed higher viral loads in sputum as compared to mild-to-moderate individuals. In addition, the probability of progression to severe disease was positively associated with sputum viral loads at baseline [28]. As noted in the explanatory box (Table 1), the majority of the publications cited here utilized quantitative reverse transcription polymerase chain reaction (RT-qPCR) and adopted the Ct value or quantified copy number as a benchmark for viral load. However, given that RT-qPCR cannot differentiate infectious from non-infectious SARS-CoV-2, no reliable conclusions can be drawn regarding an individual's infectious status.

SARS-CoV-2’s main replication sites include ciliated cells of the URT [29] and pneumocytes and macrophages of the LRT [30]. However, leakage of SARS-CoV-2 virus from (damaged) alveolar cells at the LRT to the blood circulation or direct infection of endothelial cells lining the blood circulation are hypothesized to drive SARS-CoV-2 viremia, subsequently leading to systemic spread [31,32]. Given the correlation between LRT viral load and disease severity, it is biologically plausible that a comparable correlation exists in plasma. This relation has been confirmed by studies that have demonstrated the plasma viral load of hospitalized severe patients to correlate with 90-day all-cause mortality [23] or to be linked to an increased likelihood of mortality [24]. It is important to note that most studies cited in this section examined viral loads in patients infected at the time the SARS-CoV-2 wild-type strain (Wuhan-Hu-1) was dominant. Exceptions include studies conducted from March 2020 to April 2021, during which both the Wuhan-Hu-1 and Alpha variants were prevalent [19,22], and one study from March 2020 to January 2022 [24], including various variants. Different variants, such as Omicron, which has reduced LRT replication competence compared to the Wuhan-Hu-1 and Delta strains [33], exhibit distinct viral dynamics. These dynamics may also be influenced by the individual's vaccination status [34,35]. Thus, the provided overview should be interpreted with caution in the context of current variants.

It is interesting to note that viremia is not limited to severe or critically ill patients, as asymptomatic [25] and moderate patients [26] also display viremia, although typically with lower than average loads (Figure 1). We hypothesize that individuals who exhibit viremia but undergo mild illness experience a delayed robust initial immune response, and over time, their immune systems may effectively reduce viral replication and thereby prevent disease progression.

To summarize, hospitalized patients with increased URT, LRT, and plasma viral load show more severe disease and increased mortality [14,17,18,19,22,23,24,25,26]. It should, however, be noted that most research on SARS-CoV-2 viral loads in the LRT and plasma and, to a lesser extent, the URT, primarily focuses on hospitalized or ICU patients. This group often reflects severe clinical cases. Furthermore, non-hospitalized individuals experiencing mild symptoms during the acute phase are also at risk of developing PASC [3]. Therefore, it is intriguing to map the viral load in the LRT and plasma of asymptomatic and moderate patients, as data from such longitudinal studies could shed light on its potential as an early prognostic indicator for severe COVID-19 and PASC development. The next section will elaborate on research that investigated differences and overlap between the viral loads observed during acute and PASC disease.

### 3.2. Relation between Acute-Phase Viral Dynamics and PASC Phase

While most SARS-CoV-2 positive individuals typically exhibit a detectable viral load for a brief period, initial case report studies during the pandemic showed persistence of viral RNA and infectious SARS-CoV-2 in patients who experienced prolonged symptoms. For instance, nasopharyngeal swabs from a non-hospitalized person experiencing mild acute infection remained positive, even after 110 days following the initial test. Considering the infection period from June to December 2020, we assume that she was infected with the Wuhan-Hu-1 strain. During this period, she experienced cardiovascular, respiratory, and musculoskeletal PASC-related symptoms [36]. Furthermore, SARS-CoV-2 RNA was detected in the cerebrospinal fluid 114 days after the initial positive nasopharyngeal swabs (first positive test: October 2020) in an individual experiencing prolonged central nervous system (CNS)-related symptoms [37]. Given the initial positive test in October 2020, she most likely contracted the Wuhan-Hu-1 strain, though reinfection with the Alpha variant is possible since it appeared in early 2021 in Czech Republic [38]. A similar trend was revealed in a cohort study, which showed that plasma from a significant portion of vaccinated PASC patients (60%) with ≥ three co-morbidities and who experienced mild/moderate acute COVID-19 remained positive for SARS-CoV-2 RNA over a median follow-up period of 2 years, in contrast to their matched controls (8%) [39]. Moreover, plasma of PASC patients were more frequently found to test positive and displayed higher average viral loads as compared to previously infected persons without PASC [40]. These and other case or cohort studies have addressed the need for longitudinal studies that monitor viral loads over an extended period. 

Symptoms of PASC, characterized by memory-related issues, were associated with early SARS-CoV-2 plasma viral load levels in patients with mild-to-critical acute COVID-19 (Table 2) [41]. However, this association was not observed for other PASC subtypes, including anosmia and fatigue. A longitudinal study monitoring hospitalized, severely/critically diseased patients showed that persons with viremia at time of hospital admission were more likely to experience PASC symptoms at 6 months (± 2 months) as compared to hospital-admitted patients without viremia (Table 2) [42]. Similar observations were made by Ram-Mohan et al., who noted that patients who exhibited viremia at baseline were more likely to develop PASC-related symptoms, particularly among those with moderate disease [43]. Furthermore, it was found that a low Ct value in URT swabs, e.g., high viral load (Table 1), was associated with a greater number of PASC-related symptoms (Table 2) [44]. 

What could be the biological rationale behind the increased likelihood of individuals who experienced viremia during the acute phase to develop PASC symptoms? We hypothesize that viremia, potentially leading to systemic dissemination, might result in the infection of multiple organs distant from the initial site of infection, leading to pathophysiological damage and subsequent manifestation of PASC symptoms. However, it is imperative to underscore that the presence of viremia and its subsequent inflammatory response often correlate with severe clinical presentations of acute infection, and the risk of developing PASC is higher among severe cases [45]. Therefore, we also suggest that disease severity may introduce a confounding factor in this context.

Although it was shown that RNA persists in various parts of the bodies of PASC patients [36,37,46], the detection of persistent RNA is generally assessed by PCR (Table 1). Therefore, it remains inconclusive whether the detected persistent RNA originates from RNA remnants or infectious virus. However, considering that RNA is degraded relatively quickly in the human body [47], it is unlikely that the detected RNA concerns remnants of infectious virus originating from the acute phase. Hence, several alternative theories have been proposed to explain RNA persistency, including reverse-transcribed parts of SARS-CoV-2 RNA that are integrated into the host genome, although additional studies are needed to elucidate this theory [40,48]. Moreover, the presence of infectious virus in immune-privileged sites, including the CNS, a mechanism used by other RNA viruses, e.g., Ebola virus and poliovirus [49], may also provide a plausible reason for RNA persistence in PASC patients. 

Reports on viral loads in PASC patients primarily focus on plasma samples and, to our best knowledge, limited data are available regarding the relationship between URT and LRT viral loads during acute disease and PASC development. Considering that a notable proportion of PASC patients merely experienced mild acute infection [3], it is interesting to further study viral loads in different body compartments, e.g., LRT and URT, in patients who have experienced mild acute COVID-19. Moreover, not all PASC individuals exhibit sustained viral loads, and thus, viremia is not likely to constitute an unambiguous predictor for PASC development. Yet, the summarized findings indicate that a significant portion of PASC persons remains with detectable viral loads, which could be the direct cause or related to sustained inflammation that is often observed in PASC patients, which will be elaborated on elsewhere in this review. Nevertheless, it remains interesting to speculate about the correlation between viral load and the likelihood of experiencing persistent infection. For instance, it is conceivable that a brief period of high viral load, perhaps just hours, could enable the virus to penetrate various organs and consequently establish persistency. Conversely, it is plausible that persistence merely occurs when an individual maintains a high viral load over an extended period, such as weeks.

## 4. Cytokines and Chemokines

### 4.1. Cytokines and Chemokines during Acute COVID-19

**Figure 2:**
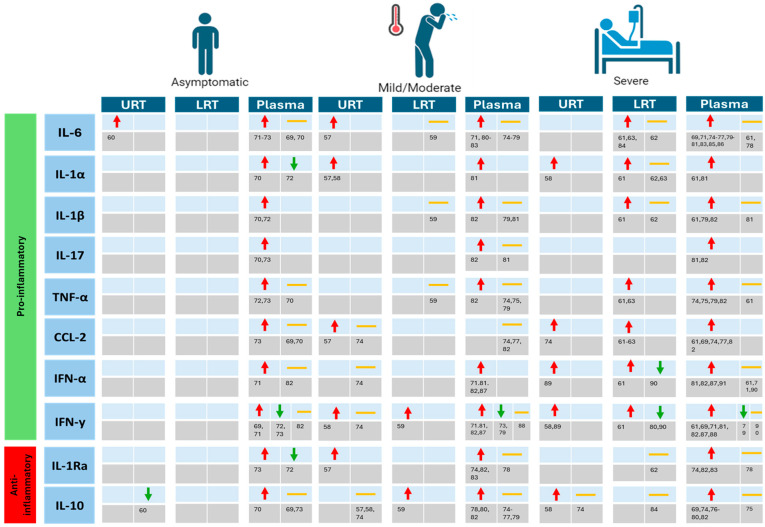
Overview of significant differences in levels of cytokines and chemokines in SARS-CoV-2-positive patients with various clinical acute-phase classifications as compared to healthy, non-infected controls. Red arrow (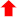) indicates a significant increase in cytokine or chemokine levels as compared with healthy controls. Orange middle line (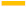) indicates no significant difference in cytokine or chemokine levels as compared with healthy controls. Green arrow (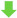) indicates a significant decrease in cytokine or chemokine levels as compared with healthy controls. Empty tables implicate that no literature regarding this cytokine or chemokine in the context of the clinical classification and sample location could be found. References used include [57,58,59,60,61,62,63,69,70,71,72,73,74,75,76,77,78,79,80,81,82,83,84,85,86,87,88,89,90,91], and specific references are found in the corresponding part of the figure.

Disease severity progression is not solely dictated by viral loads, but is, rather, influenced by the host’s response to these viral loads [50]. This complex interplay, referred to as host–pathogen interaction, involves numerous processes, including secretion of cytokines and chemokines initiating a comprehensive immune response, facilitating viral eradication. Given the vast number of cytokines and chemokines that are released during SARS-CoV-2 infection, a particular subset was chosen (Figure 2). This selection was not exclusively because of its frequent mentioning in the literature, but also because the individual cytokines and chemokines play distinct roles in the immune response. For example, IL-17 functions as an inflammation mediator frequently observed in autoimmunity [51], whereas IL-6 is a pleotropic cytokine reported to play a crucial role in viral infections [52]. Furthermore, IL-10 is recognized for its protection from immune-mediated damage [53], whereas TNF-α is involved in initiating tissue necrosis or apoptosis and promoting lung fibrosis [54]. Considering PASC, where lung fibrosis is frequently mentioned [55], highlighting a range of functionally divergent cytokines is important. Lastly, interferon-α and -γ (IFN-α and IFN-γ) were included given their multifunctional role in the frontline defense against viral infections [56].

Reports have shown elevated release of both pro- and anti-inflammatory cytokines in the nasopharynx of patients with varied clinical classifications of COVID-19 disease [57,58,59]. While there is a scarcity of studies examining asymptomatic patients, Xie et al. concluded that the URT of unvaccinated, asymptomatic patients infected with Wuhan-Hu-1 is characterized by elevated levels of pro-inflammatory IL-2 and IL-6 and decreased levels of anti-inflammatory protein IL-10 [60]. For patients with moderate and severe COVID-19, a higher variety of pro- and anti-inflammatory cytokines and chemokines in the URT was reported to be elevated as compared to their healthy controls (Figure 2). However, this may be biased due to the existence of more reports that have investigated the immune environment of the URT in symptomatic patients as compared to asymptomatic patients. Nevertheless, the overall trend is that increased release of cyto- and chemokines in the URT has been measured in all COVID-19 disease types, and therefore, it appears to be independent of the COVID-19 disease status.

Given that disease severity increases when viral infection extends to the LRT, it is intriguing to investigate whether the cyto- and chemokine profile at this anatomical site is more common in severe COVID-19 cases or independent of the disease status. In patients with moderate COVID-19 disease, only IL-10 levels were found to be increased in the LRT as compared to healthy controls whereas levels of IL-6, IL-1β and TNF-α remained unchanged [59]. In contrast, both the variability in function and the number of upregulated cyto- and chemokines increased with disease severity (Figure 2). This indicates a broader inflammatory state in the LRT of patients with severe disease. Here, results regarding cytokines and chemokines within the LRT of patients with SARS-CoV-2 infection are mainly based on articles that investigated mild/moderate or severe patients predominantly infected with Wuhan-Hu-1 or Alpha variants [59,61,62,63]. However, as previously mentioned, LRT replication of SARS-CoV-2 seems to vary by variant [33]. Future studies should thus explore whether the cytokine signature in the LRT of patients infected with various variants, including Delta and Omicron, displays similar or distinct patterns.

In the context of viral infections, elevated cyto- and chemokines in plasma indicate severe disease. This has been observed for severe cases of viral respiratory diseases, including MERS, SARS, and highly pathogenic Influenza H7N9 [64,65,66]. COVID-19 disease does not deviate from this pattern, as elevated plasma cyto- and chemokine levels were more often detected in patients with severe COVID-19 disease (Figure 2). Interestingly, elevated levels of IL-1α, IL-1β, and CCL-2 were observed in the plasma of severe COVID-19 patients [61]. However, in the same patients, BAL analysis revealed that, in addition to the aforementioned cytokines, IL-6 and TNF-α were also significantly higher in severe patients as compared to healthy controls. This illustrates that, in the LRT, an even higher variety of elevated inflammatory mediators is present as compared to plasma. We postulate that this may be attributed to the intricate LRT immune environment, which could lead to a wider array of cytokines and chemokines in response to viral infection compared to those found in plasma, primarily reflecting release from other parts of the body. As enhancers of the early antiviral response, levels of IFN-α and IFN-γ were found to be elevated at both the URT and LRT and in the plasma of patients across various clinical scores (Figure 2). In fact, research has demonstrated the importance of exhibiting a robust early IFN-γ response, as it has been found that low plasma levels of IFN-γ at symptom onset serve as a predictive marker for severe disease, especially when adjusting for factors such as vaccination and comorbidities [67]. Moreover, unvaccinated, fatal COVID-19 cases were characterized by a weak early IFN-γ response in the URT [58]. Thus, in the clinical context, it is of importance to collect samples at the onset of symptoms if IFN-γ is to be used as a biomarker for predicting disease progression and potentially guiding clinical interventions with IFN modulators.

Furthermore, reduced inflammatory levels, including IFN-α and IFN-γ, were found in the plasma of vaccinated as compared with unvaccinated symptomatic individuals [68]. Thus, these results suggest that vaccination may contribute to preventing the excessive inflammatory response often seen in patients with severe disease [20,68].

In conclusion, although the cytokine and chemokine response within the URT appears to be independent of clinical presentation, its release in distant areas from the initial infection site, e.g., LRT and plasma, is associated with disease severity. Determining whether this association aligns with the viral load in these regions may clarify the factors influencing disease progression.

### 4.2. Relationship between Viral Load and Cyto- and Chemokine Markers across COVID-19 Disease Subsets

Pathophysiological consequences of viral infections are not solely due to viral replication, but additionally arise from an often dysregulated immune response. Therefore, considering predicting clinical progression, this section elaborates on the question of whether correlations between viral load and release of cyto- and chemokines exist and how these may vary across clinical spectra of acute-phase disease.

Study results on non-vaccinated Wuhan-Hu-1-infected individuals demonstrated a positive correlation between URT viral loads and plasma cytokines IL-6 and IL-10, but not with TNF-α and INF-γ (Table 3) [76]. Furthermore, research showed that in unvaccinated, Wuhan-Hu-1-infected individuals, only plasma CCL-2 levels significantly correlated with URT viral load, whereas such a correlation was absent for a large number of other cytokines and chemokines (Table 3) [77]. In another study investigating a panel of cytokines and chemokines, including IL-6, IL-1α and IL-1β, IL-17, TNF-α, IL-1Ra, IL-10, and CCL-2, only plasma levels of CCL-2 correlated with plasma viral load in unvaccinated, symptomatic patients with confirmed Wuhan-Hu-1 SARS-CoV-2 infection (Table 3) [82]. Nevertheless, it is important to note that these studies did not include stratification based on disease severity.

Research that applied disease stratification has found the plasma, URT, and LRT viral loads of hospitalized, unvaccinated individuals to be correlated with various plasma cytokines, including IL-6, IL-8, and CCL-2 [32]. However, this association was not present for numerous other cytokines and or chemokines including IFN-γ and IL-1RA. Therefore, the authors emphasized that, although viral load could be a direct contributor to the elevated inflammatory markers at several sites in the body, the observed elevated inflammation could be multifactorial. Lastly, Smith et al. showed that, in patients with mild-to-moderate or severe-to-critical disease, the plasma viral load was positively associated with plasma-located TNF-α, IL-6, and anti-inflammatory proteins IL-10 and IL-1RA [74]. On the contrary, URT viral loads were negatively correlated with IFN-γ and IL-33 levels located in the URT (Table 3) [74].

Collectively, there are indications of correlations between viral load and cytokines in the URT, LRT, and plasma of patients with COVID-19. Nevertheless, the lack of stratification based on disease severity impedes the ability to forecast elevations of cytokines and/or chemokines based on viral load. Another limitation is that the data provided herein are based on studies that primarily included patients infected during periods when the Wuhan-Hu-1 strain was predominant.

### 4.3. Differences and Similarities in Cytokine Profiles in Acute and PASC

Given the diverse range of symptoms associated with PASC, various processes such mitochondrial energy production and latent virus reactivation may play a role in symptom manifestation [4]. Within these processes, cytokines and chemokines are consistently identified as important factors, and thus, investigating the cytokine and chemokine profiles of PASC patients has become relevant. Schultheiẞ et al. demonstrated significant elevations of TNF-α, IL-1β, and IL-6 in PASC patients as compared to previously infected persons without PASC [92]. Within this study, the majority of the individuals were vaccinated (>73%) and primarily experienced mild/moderate COVID-19. However, their results regarding cytokine elevations were not corrected for parameters such as vaccination status or SARS-CoV-2 variants [92]. Likewise, PASC individuals with unreported vaccination statuses demonstrated a 44% elevation in IL-6 levels compared to control subjects without PASC [93]. The majority (84%) of concerned PASC patients who were not hospitalized during acute COVID-19 infection and at least 42% of the included persons displayed at least one comorbidity. Moreover, the highest increase was observed in PASC patients characterized by cardiopulmonary complaints, which demonstrated the degree of IL-6 elevation to vary across PASC disease phenotypes. In another study, IL-6 levels in unvaccinated, Wuhan-Hu-1-infected PASC patients were not statistically different as compared to control subjects without PASC 8 months following initial infection [94]. This contradicting finding is of particular interest as the above studies consisted of PASC patients with comparable clinical conditions, namely, mild-to-moderate acute illness. This emphasizes the heterogeneity of the results regarding the (remained) elevation of specific cytokines in the PASC phase.

**Figure 3:**
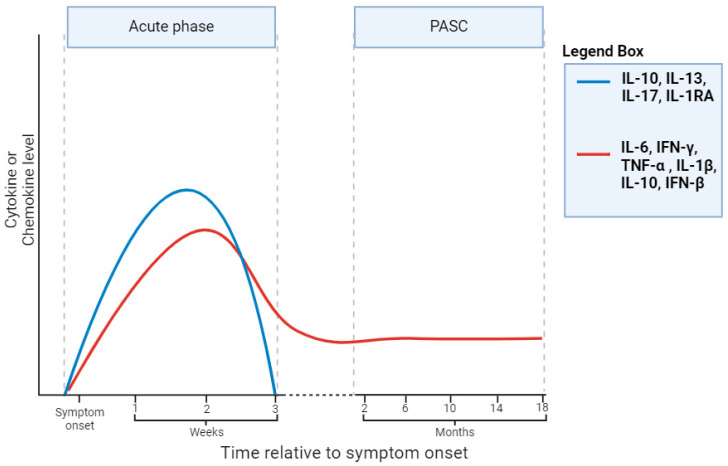
Graphical representation of cytokines and chemokines during acute phase of COVID-19 disease and subsequent PASC phase. Blue line indicates cytokines and chemokines for which literature evidence exists that levels increase during acute phase and diminish during PASC phase. On the contrary, red line indicates cytokines and chemokines that remain elevated during PASC phase. References: blue line: IL-10 [96,99]; IL-13 [96]; IL-17 [96]; IL-1RA [96]. References: red line: IL-6 [92,93,96]; IFN-γ [95,96]; TNF-α [92]; IL-1β [92,96]; IL-10 [98]; IFN-β [94,95]. Note that literature on IL-10 shows conflicting data. Figure created using https://www.biorender.com/ (accessed on 21 March 2024).

Findings from a longitudinal study indicated that PASC patients were characterized by significantly lower IFN responses during their acute period as compared to patients without enduring symptoms [95]. However, this IFN response persisted over time in PASC patients, whereas this was not observed in non-PASC patients. Significantly higher levels of IFN cytokines, specifically IFN-β, were found in PASC patients 8 months post-infection as compared to non-PASC patients [94]. Research conducted by Torres-Ruiz et al. highlighted significantly elevated baseline levels of IL-1α and IP-10, with a trend of higher levels of IL-6, IFN-γ, and IL-1β in patients who later developed PASC as compared to those who recovered from acute infection [96]. On the contrary, no differences were observed for IL-10, IL-13, IL-17, TNF-α, and IL-1RA (Figure 3). This is of particular interest, as we highlighted in the previous section that these cytokines and chemokines were upregulated in the plasma of moderate and severe COVID-19 patients. Therefore, this points out that only several cytokines and chemokines remained or became elevated during the weeks or months after COVID-19 infection in patients who later developed PASC.

Besides pro-inflammatory cytokines, data are available on the levels of anti-inflammatory cytokines in vaccinated PASC patients. For instance, at the metabolite level, patients displayed elevated concentrations of anti-inflammatory metabolites, although anti-inflammatory cytokines and chemokines were not investigated at the protein level during this study [97]. Another study demonstrated that the anti-inflammatory cytokine IL-10 remained elevated in unvaccinated patients suffering from severe PASC as compared to previously infected unvaccinated persons without PASC [98]. Nevertheless, these elevated levels only lasted for 2 months, followed by a smaller difference in IL-10 levels between severe PASC and non-PASC patients, indicating that IL-10 levels return to baseline over time. On the contrary, others reported lower levels of IL-10 and IL-4 in unvaccinated PASC patients as compared to unvaccinated non-PASC individuals, which suggested better control of the inflammatory process [99].

We do realize, however, that the summarized studies show conflicting results regarding the cytokine and chemokine signature in PASC patients. Such discrepancies may be attributed to confounding factors, such as differences in PASC disease phenotype, SARS-CoV-2 variants, vaccination status, comorbidities, and sex. These confounding factors are often not mentioned in all cited articles and are more likely to affect outcomes in studies with relatively small, homogenous populations than in those with larger, more diverse groups. Therefore, additional cohort studies comprising heterogenous study populations that take these confounding factors into account could give more insight into the cytokine and chemokine molecular signatures of persons who later developed PASC. However, the limited number of newly reported COVID-19 and, therefore, PASC cases impedes the set-up of such longitudinal studies. Therefore, retrospective studies using samples taken earlier may offer a solution to study the dynamics involved in PASC development.

Despite conflicting evidence, most current research points out that certain PASC patients show enduring immune activation characterized by sustained levels of pro- and anti-inflammatory cytokines and chemokines. Several cytokines that are elevated at the time of acute COVID-19 remain or become elevated during PASC. These include, but are not limited to, IL-6, IFN-γ, TNF-α, and IL-1β (Figure 3). There have been various hypotheses postulated regarding the underlying biological cause of persistent immune activation. These include viral persistency in reservoir cells, microclot formations, and re-activation of latent (herpes) viruses such as Epstein–Barr virus [100]. These hypotheses are not mutually exclusive and may vary across PASC phenotypes, or they may potentially serve as the underlying cause for the distinct described PASC phenotypes.

## 5. In Vitro Models and Preclinical Animal Models to Study Acute COVID-19 and PASC

Studying acute COVID-19 and PASC in humans enables researchers to explore the natural progression of infection; nevertheless, drawbacks exist. These include uncontrolled conditions, comorbidities, and resource-intensive procedures. Employing (preclinical) models, from in vitro co-culture systems to animal models, allows researchers to control factors and bypass most described drawbacks. For instance, studies using cultures of human peripheral blood mononuclear cells (PBMCs) from both healthy individuals and severe COVID-19 patients were conducted to investigate the viral tropism of SARS-CoV-2 [101]. Moreover, in vitro co-culture systems were utilized to examine the initial interaction between immune cells and SARS-CoV-2-infected epithelial cells. This approach allowed researchers to study the underlying factors that might contribute to immune dysregulation in acute COVID-19 [102]. More complex 3D cell culture models, such as organoids and microfluidic devices, have been explored to mimic the viral and immune dynamics of SARS-CoV-2 and serve as a preliminary approach for drug evaluation [103,104]. This approach may have the potential to circumvent the commonly observed, relatively poor translatability of drug efficacy from animal testing to human treatment [105]. Moreover, the number of animal tests for preclinical studies could additionally be reduced by employing 3D cell culture techniques.

Early in the pandemic, several preclinical animal models were quickly mapped to mimic both the acute and PASC phase. Here, we provide a brief overall of the commonly used small animal models (Table 4). This selection is based on the relative ease of utilizing small animal models as compared to non-human primates, which, in addition, presents ethical limitations. Animal models have extensively been described in other review papers [106,107], and therefore, for further information, we recommend consulting those sources. From the overview (Table 4), it can be deducted that several animal models can be employed to answer a range of COVID-19-related questions. For instance, viral replication during asymptomatic infection could be studied using the ferret model [108]. Moreover, both transgenic mice and Syrian hamsters are suitable for studying the efficacy and safety of vaccines and therapeutic drugs. For instance, in Syrian hamsters, both Molnupiravir [109] and Nirmatrelvir [110] showed potent efficacy against various variants, including Omicron. In mice, a combination of Molnupiravir and Nirmatrelvir demonstrated significant inhibition of SARS-CoV-2 replication [111].

Common limitations exist in the currently available in vitro and preclinical models for COVID-19 related research. For instance, the primary health burden of COVID-19 predominantly originates from patients experiencing severe symptoms, often in the presence of comorbidities. Most animal models, however, only display mild disease, with limited reports investigating comorbidities and their effects on acute-phase disease. When it comes to PASC, similar trends apply. Although Syrian hamsters showed PASC-related behavioral changes [112], an animal model that adequately reflects human PASC symptoms remains absent. Moreover, susceptible animal models supporting a long duration of virus replication and competent models to study different hypotheses underlying the molecular mechanisms that drive PASC are still missing. Given that PASC is primarily characterized by behavioral impact, 3D cell culture methods may have limited added value in this area, and thus, animal models remain the preferred choice to study PASC. The various techniques available, from co-culture systems to animal models, should be employed to address the gaps and limitations inherent in each method. In addition, future developments in animal models and 3D cell culture methods may overcome the identified limitations, offering potential benefits for both preclinical studies and therapeutical approaches in the future.

**Table 4:** Common animal models and their findings in relation to acute COVID-19 and PASC.

Animal	Susceptibility	Severity	Acute Phase	PASC	Ref.
Transgenic Mice	Upon hACE2 expression Use of mouse adapted strains	Mild Severe	K18-hACE2 mice: hACE2 overexpression results in systemic virus spread and mortality. Display lethargy and edema-associated acute lung injury, similar to ARDS. High titer infection results in upregulation of various cytokines in lung and brain at 7 dpi.	Mouse-adapted strain induced neuropathological changes in BALB/c mice. May resemble cognitive dysfunction PASC symptom.	[113,114]
Syrian Hamsters	Natural	Mild Severe	Increases in various cytokines. High viral loads of 2–8 dpi. Undetectable after ~10 days post infection (dpi). Inoculum volume impacts severity. Late and weak immune influx and prolonged weight loss in aged hamsters. Diet-induced obese hamsters show sustained inflammation and may resemble humans with obese comorbidity.	Sustained inflammation in olfactory tissue (31dpi); may resemble PASC-like behavior symptoms in humans. Anosmia caused by olfactory epithelial damage.	[112,115,116,117,118]
Ferrets	Natural	Asymptomatic Mild	Intranasal challenge results in asymptomatic infection with URT virus replication. Used for transmission studies. Infection of primarily URT for 8 dpi post-infection with low mortality. Viral shedding in nasal, saliva, urine, and fecal samples until 8 dpi. Aged ferrets display higher viral loads, prolonged nasal virus shedding, and severe lung inflammatory cell infiltration. Used for evaluation of vaccine and antivirals.	Minimal reported resemblances with human PASC. Indications for sustained pathological URT abnormalities potentially reflecting long-term impact of infection.	[107,108,118,119]

## 6. Concluding Remarks and Future Directions

The health, economic, and societal burden of PASC underscores the need to investigate the underlying mechanisms and factors that drive PASC development. Given that PASC is a multifactorial condition with diverse underlying mechanisms, comprehensive understanding and progress in addressing this complex disease necessitate collaborative efforts across various disciplines. Only through translational research integrating insights from clinical, biological, and epidemiological domains can meaningful advancements be made towards unraveling the complexities of PASC. Here, we merely deliver a virologist’s perspective in light of available data on the differences in viral loads and cytokine and chemokine responses during the acute and PASC phases. We emphasize that the likelihood of experiencing severe or critical progression increased with viral load levels detected in the URT, LRT, and plasma of hospitalized patients. Acute-phase viremia is a well-characterized, but not unambiguous, predictor of PASC development. Viral RNA, either from infectious virus or remnants, persists in persons that experience PASC. Cytokine and chemokine levels vary significantly across acute-phase clinical conditions. Both the quantity and diversity of functions increase with disease severity. Comparing the acute phase and PASC phase showed that specific cytokines remain or become elevated in the PASC phase. This ongoing inflammation may be the driving factor behind enduring symptoms. The driving factor of ongoing inflammation, whether it be persistent infectious virus hiding in immune-privileged sites, remnants of non-infectious virus, re-activation of latent viruses, or a combination of the potential causes, currently remains unknown.

## Figures and Tables

**Table 1:** Explanatory box with commonly used terms and their corresponding definitions as used here in this review.

Term	Description
PASC or Long COVID	WHO definition of PASC is adopted for this review. This defines PASC as the continuation or development of new symptoms 3 months after the initial SARS-CoV-2 infection, with these symptoms lasting for at least 2 months with no other explanation.
Acute phases spectrum	The phases, asymptomatic, mild, moderate, severe and critical are most often used. However, in this review, the spectrum includes asymptomatic, mild/moderate and severe phase. Rationale for this deviation is that is the review focuses on the differences and progression from non-severe phases (asymptomatic, mild/moderate) to the severe phases.
Asymptomatic	Individuals positive for COVID-19 in absence of infection related symptoms.
Mild/Moderate	Individuals who display mild symptoms including fever, muscle pain and anosmia and moderate symptoms including tachypnea and mild pneumonia without the need for hospitalization.
Severe	Hospitalized patients with clinical signs of pneumonia, severe tachypnea, severe dyspnea and critical symptoms including respiratory and organ failure and coma.
Cytokine storm	State of deregulated immune system, characterized by an excessive production and release of cytokines and chemokines. Release may cause damage to various tissues including lung. Cytokine storm may subsequently trigger acute respiratory stress syndrome leading to organ failure or even death.
Viral load	Commonly quantitatively expressed as concentration in viral copies/mL in plasma. Here, viral load magnitude is defined by the copy number or Ct value determined via (RT-q)PCR. This definition is widely used in the papers cited in this review and is therefore adopted here.
Viremia	Classically defined as the presence of (non-) infectious whole virus or immune neutralized whole virus in serum. Publications referred to here do not use viral culture or staining to determine presence of virus in serum. In turn, the majority uses (RT-q)PCR and adopt the Ct value or quantified copy number as a benchmark for viremia. Therefore, in case the review refers to viremia or viral load, remember that these are Ct values representing RNA copy numbers.
Ct value	Cycle threshold (Ct) value in PCR is the number of cycles needed to indicate that a sample contains the target RNA or DNA. The Ct value negatively correlates with the initial amount of target RNA or DNA, i.e., in this review a low Ct value indicates a high viral load.
Persistent infection	Prolonged presence of SARS-CoV-2 (non-)infectious virus or RNA remnants that is detected for an extended period beyond acute infection. Persistence could be associated with display of symptoms or in absence of symptoms.
URT & LRT	Upper respiratory tract (URT) that includes nasal cavity, pharynx and larynx. Lower respiratory tract (LRT) that includes trachea and lungs.
RNA Load	Copy number of SARS-CoV-2 RNA, detected by (RT-q)PCR on samples from various origin including serum, BAL, nasopharyngeal swabs.
Inoculum	The virus quantity or concentration introduced at the time of host infection.

**Table 2:** Summary of cohort studies on relation between acute-phase viral loads and subsequent PASC development.

Study Population Characteristics	Acute-Phase Viral Load Correlation or Association with PASC Phase	Ref.
Hospitalized (*n* = 47) Vaccinated Strain variant unknown	Plasma of PASC patients tested positive more frequently (55% vs. 29%) and had higher average viral loads compared to previously infected persons without PASC. In PASC-positive patients, plasma viral load remained unchanged or increased, while in PASC-negative individuals, it decreased or became undetectable over time.	[40]
Mix of mild-to-critical patients. Exact numbers unknown. Unvaccinated * Wuhan-Hu-1 strain	Detectable plasma viral load was associated with memory-related issues, whereas there were no associations with anosmia or fatigue.	[41]
Hospitalized (*n* = 129) Severe/Critical (≥90% pneumonia)	Viremia at time of hospitalization resulted in a higher chance of PASC symptom development as compared to hospital-admitted patients without viremia.	[42]
Mild/moderate (*n* = 119) Severe/Critical (*n* = 8) Unvaccinated * Wuhan-Hu-1 strain **	Patients with detectable plasma viral load at enrollment were more likely to report PASC symptoms one month after confirmed infection as compared to individuals without detectable plasma viral loads (83% vs. 41%).	[43]
Mild/moderate (*n* = 70) Severe/Critical (*n* = 6) Vaccination status *** Strain variant unknown	Viral URT loads correlated with an increased number of experienced PASC symptoms.	[44]

* Unvaccinated given that the data of the study were published before the world initiated COVID-19 vaccination campaigns (14 December 2020). ** Given the recruitment period of March 2020–December 2020, we assume that individuals were infected with Wuhan-Hu-1. *** Article did not disclose vaccination status of enrolled individuals. Patient enrollment overlapped with ongoing vaccination campaigns.

**Table 3:** Correlations of SARS-CoV-2 viral loads with markers of inflammation.

Study Population Characteristics *	Viral Load	Cytokine		Ref.
	Origin	Origin	Correlation with ***	
Non-stratified ** Mild/Moderate (*n* = 26) Severe/critical (*n* = 11)	URT	Plasma	IL-6, IL-10	[76]
			TNF-α, INF-γ	
Non-stratified ** Asymptomatic (*n* = 6) Mild/Moderate (*n* = 17) Severe/critical (*n* = 8)	URT	Plasma	CCL-2	[77]
			IFN-α, IFN-γ, TNF-α, IL-1β, IL-2, IL-6, IL-7, IL-8, IL-10, IL-12p70, IL-13, IL-17A	
Non-stratified ** Asymptomatic (*n* = 4) Mild/Moderate (*n* = 58) Severe/critical (*n* = 8)	URT	Plasma	CCL-2, VEGF, G-CSF	[82]
			IL-1β, IL-1ra, IL-2, IL-2Rα, IL-6, IL-7, IL-8, IL-9, IL-10, IL-13, IL-15, IL-17, IL-18, IFN-α2, IFN-γ, TNF-α Whole list; see reference	
Hospitalized Severe/critical (*n* = 88)	Plasma	Plasma	IL-6, IL-8, IP10, CCL-2	[32]
			IFN-γ, IL-1RA	
	URT	Plasma	IL-6, IL-8, IP10, CCL-2, IFN-γ	
			IL-1RA	
	LRT	Plasma	IL-6	
			IL-8, IP10, CCL-2, IFN-γ, IL-1RA	
Non-stratified ** Mild/moderate (*n* = 15) Severe/critical (*n* = 34)	Plasma	Plasma	IL-6, CCL-2, CCL-19	[74]
			IFN-α2	
	URT	URT	IFN-γ, IL-33	
			IL-10	

* All patients included in the cited studies were unvaccinated. Given the recruitment period of March 2020–December 2020, we assume that they were infected with the Wuhan-Hu-1 strain. ** Non-stratified = Patients with various clinical classifications (asymptomatic, mild/moderate, severe/critical) were analyzed as a single group. *** Colored table indications: green = positive correlation; orange = no correlation; red = negative correlation.
